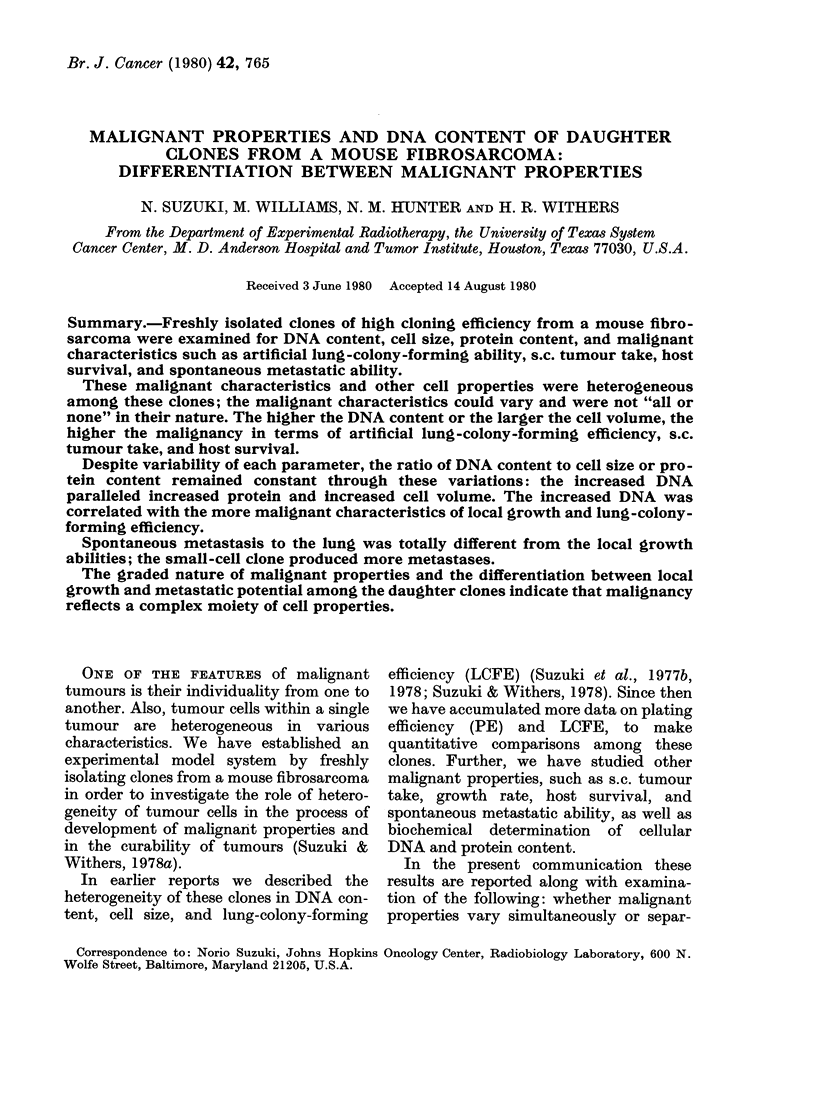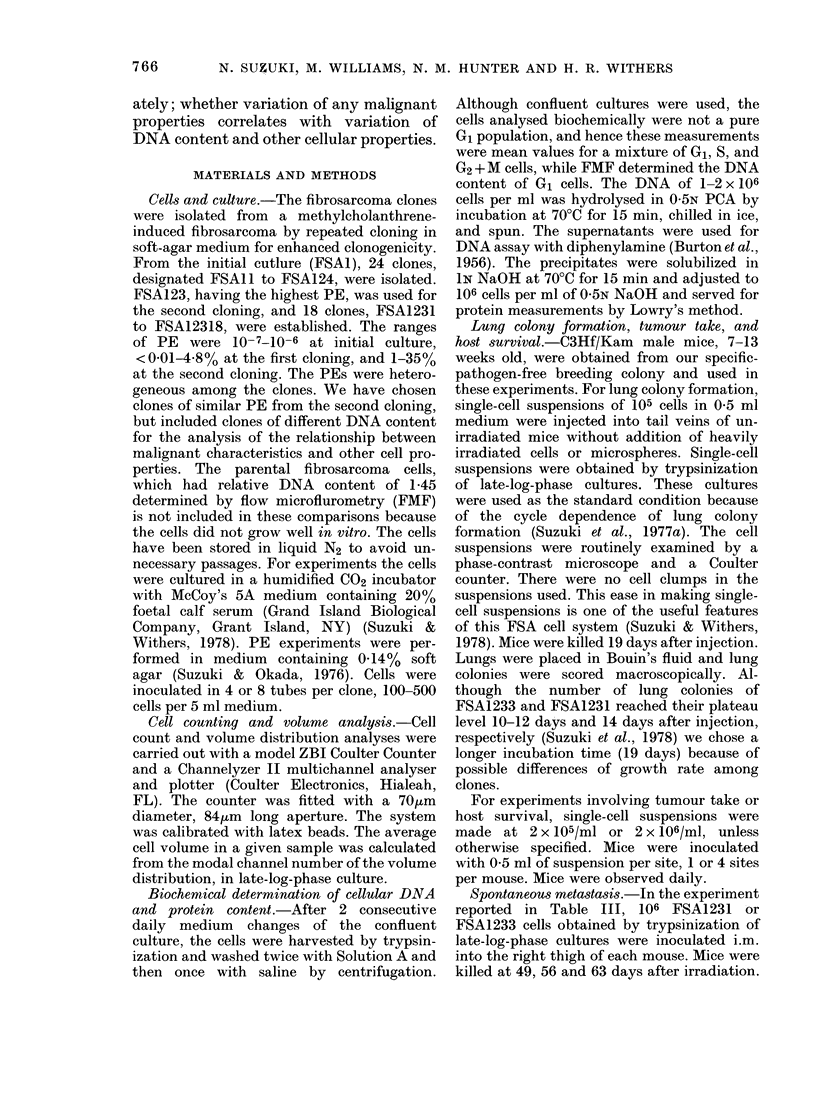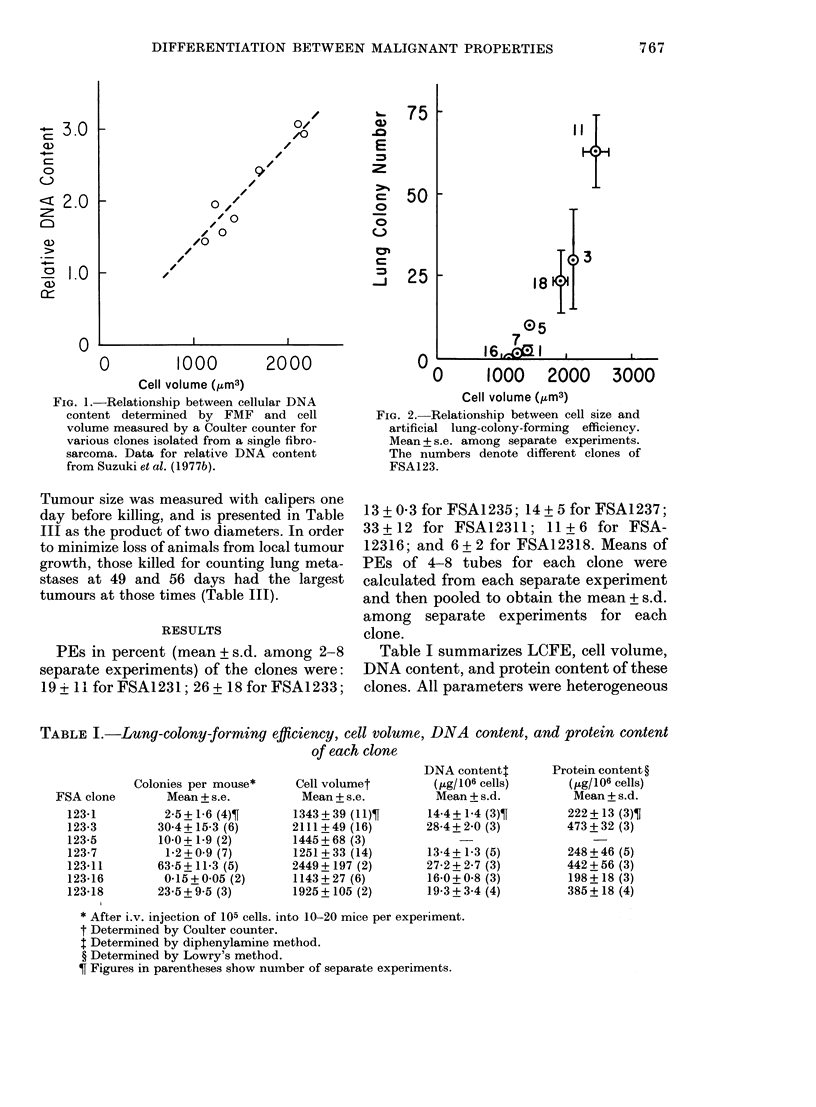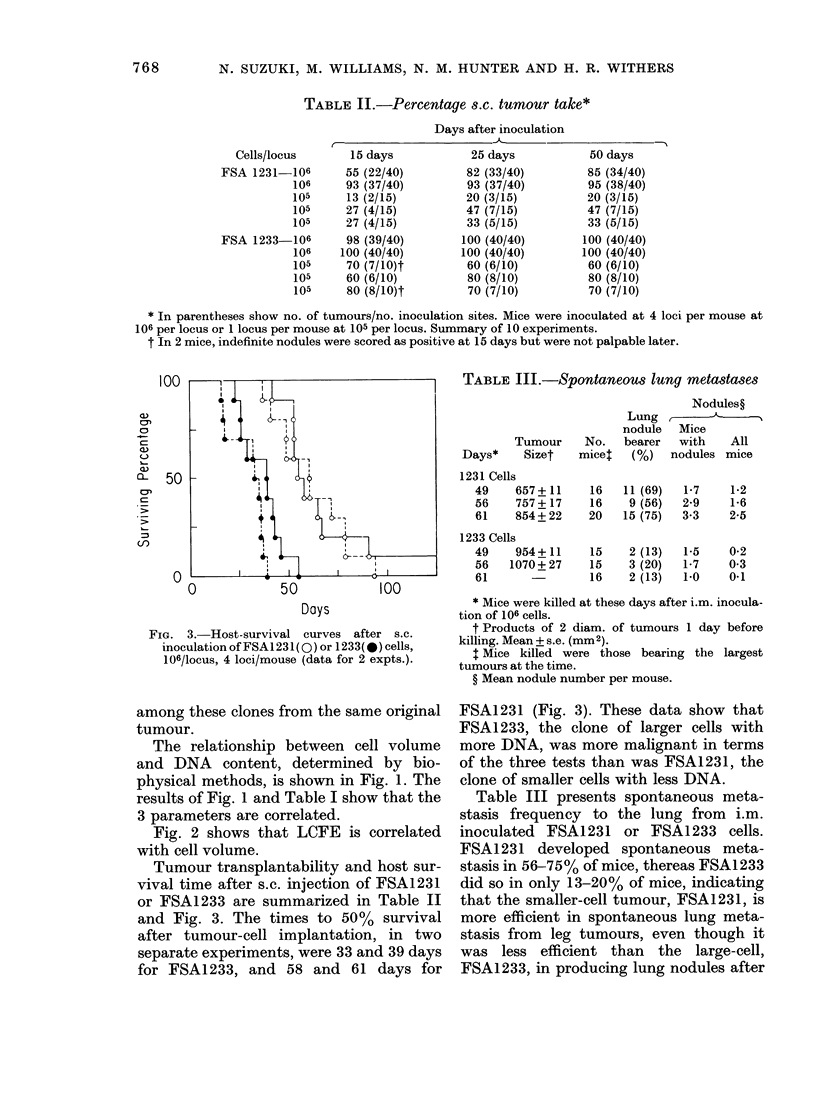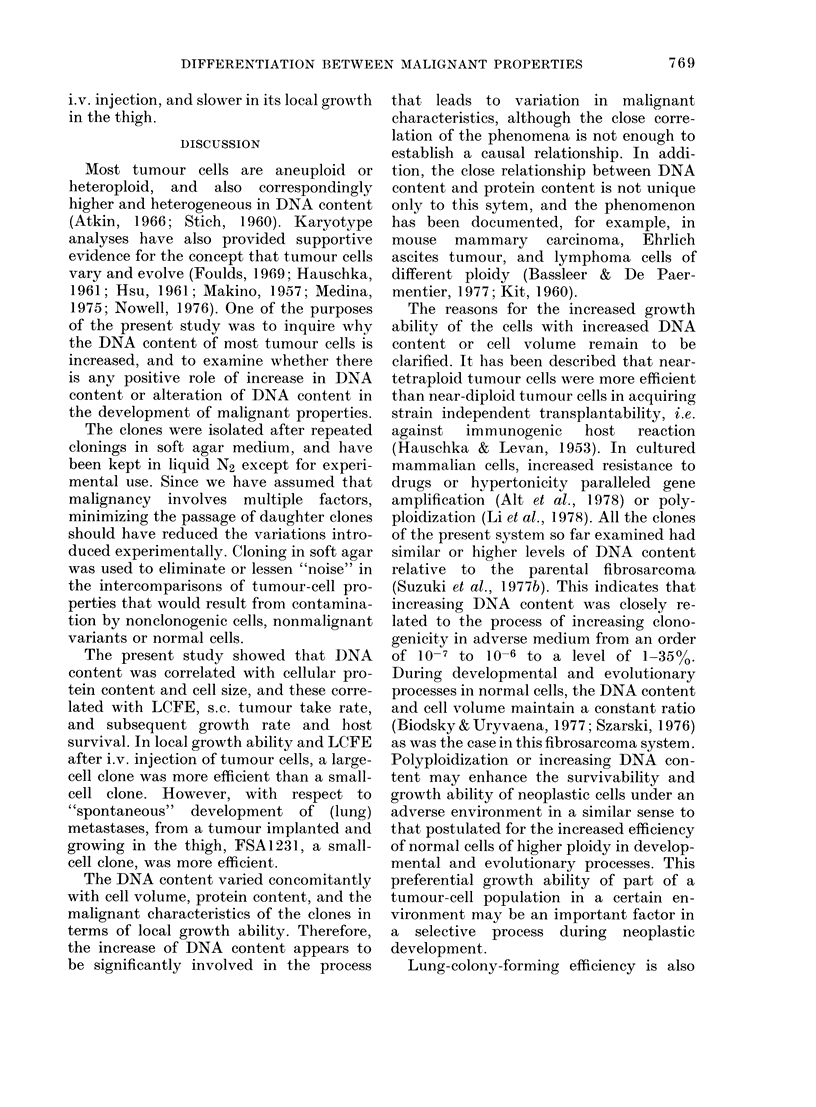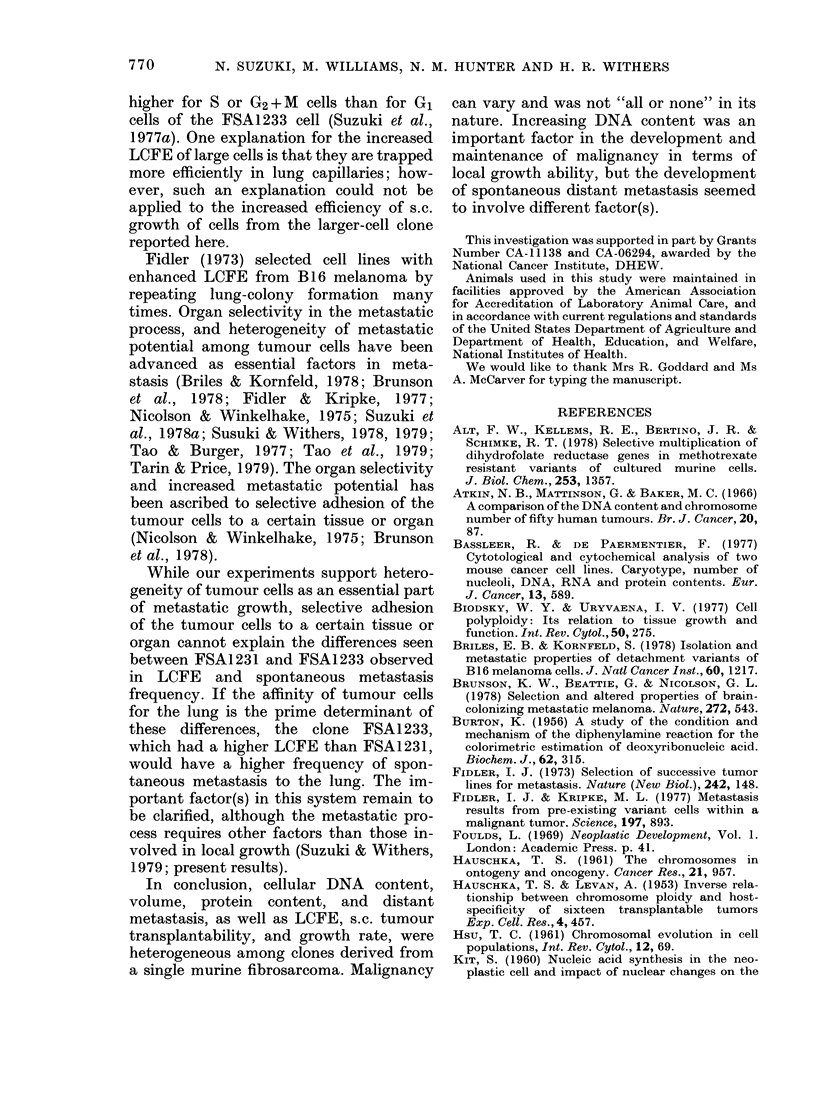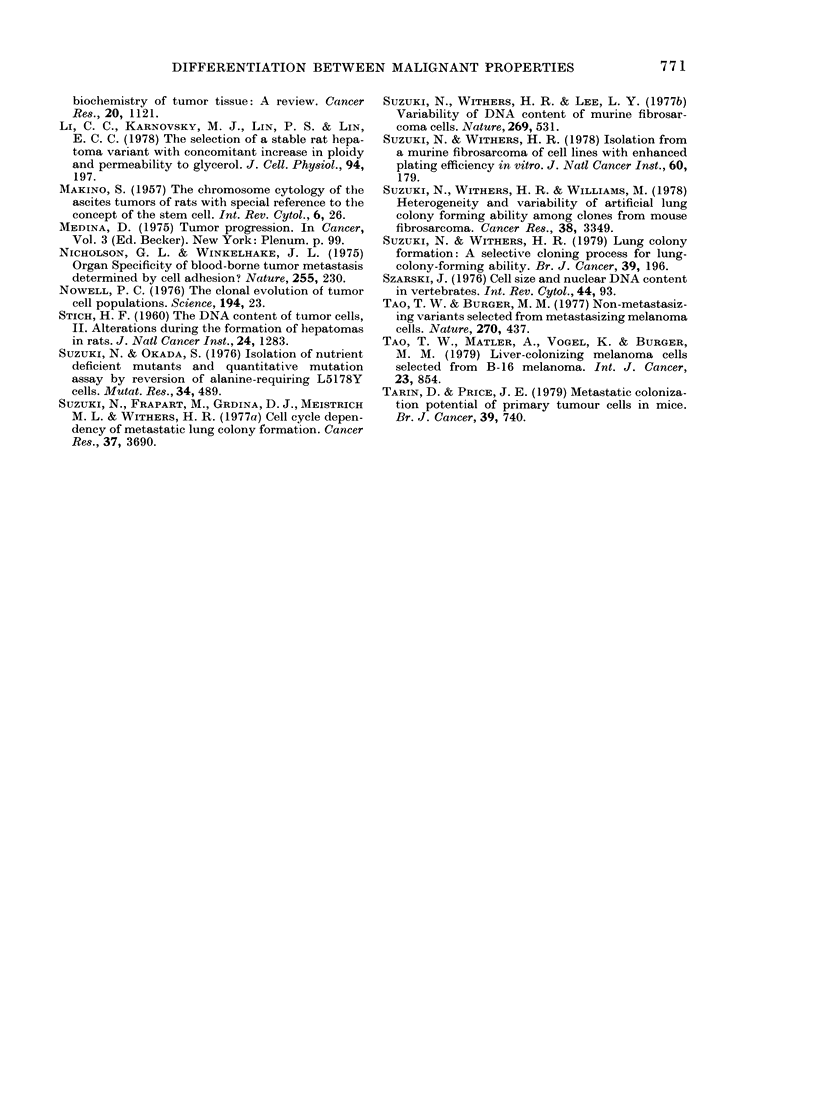# Malignant properties and DNA content of daughter clones from a mouse fibrosarcoma: differentiation between malignant properties.

**DOI:** 10.1038/bjc.1980.310

**Published:** 1980-11

**Authors:** N. Suzuki, M. Williams, N. M. Hunter, H. R. Withers

## Abstract

Freshly isolated cones of high cloning efficiency from a mouse fibrosarcoma were examined for DNA content, cell size, protein content, and malignant characteristics such as artificial lung-colony-forming ability, s.c. tumour take, host survival, and spontaneous metastatic ability. These malignant characteristics and other cell properties were heterogeneous among these clones; the malignant characteristics could vary and were not "all or none" in their nature. The higher the DNA content or the larger the cell volume, the higher the malignancy in terms of artificial lung-colony forming, efficiency, s.c. tumour take, and host survival. Despite variability of each parameter, the ratio of DNA content to cell size or protein content remained constant through these variations: the increased DNA paralleled increased protein and increased cell volume. The increased DNA was correlated with the more malignant characteristics of local growth and lung-colony-forming efficiency. Spontaneous metastasis to the lung was totally different from the local growth abilities; the small-cell clone produced more metastases. The graded nature of malignant properties and the differentiation between local growth and metastatic potential among the daughter clones indicate that malignancy reflects a complex moiety of cell properties.


*Br. J. Cancer* (1980) **42**, 765

### MALIGNANT PROPERTIES AND DNA CONTENT OF DAUGHTER CLONES FROM A MOUSE FIBROSARCOMA: DIFFERENTIATION BETWEEN MALIGNANT PROPERTIES

N. SUZUKI, M. WILLIAMS, N. M. HUNTER AND H. R. WITHERS

*From the Department of Experimental Radiotherapy, the University of Texas System Cancer Center, M. D. Anderson Hospital and Tumor Institute, Houston, Texas* 77030, *U.S.A.*

Received 3 June 1980 Accepted 14 August 1980

**Summary.—Freshly isolated clones of high cloning efficiency from a mouse fibrosarcoma were examined for DNA content, cell size, protein content, and malignant characteristics such as artificial lung-colony-forming ability, s.c. tumour take, host survival, and spontaneous metastatic ability.**

**These malignant characteristics and other cell properties were heterogeneous among these clones; the malignant characteristics could vary and were not "all or none" in their nature. The higher the DNA content or the larger the cell volume, the higher the malignancy in terms of artificial lung-colony-forming efficiency, s.c. tumour take, and host survival.**

**Despite variability of each parameter, the ratio of DNA content to cell size or protein content remained constant through these variations: the increased DNA paralleled increased protein and increased cell volume. The increased DNA was correlated with the more malignant characteristics of local growth and lung-colony-forming efficiency.**

**Spontaneous metastasis to the lung was totally different from the local growth abilities; the small-cell clone produced more metastases.**

**The graded nature of malignant properties and the differentiation between local growth and metastatic potential among the daughter clones indicate that malignancy reflects a complex moiety of cell properties.**

ONE OF THE FEATURES of malignant tumours is their individuality from one to another. Also, tumour cells within a single tumour are heterogeneous in various characteristics. We have established an experimental model system by freshly isolating clones from a mouse fibrosarcoma in order to investigate the role of heterogeneity of tumour cells in the process of development of malignant properties and in the curability of tumours (Suzuki & Withers, 1978*a*).

In earlier reports we described the heterogeneity of these clones in DNA content, cell size, and lung-colony-forming efficiency (LCFE) (Suzuki *et al.*, 1977*b*, 1978; Suzuki & Withers, 1978). Since then we have accumulated more data on plating efficiency (PE) and LCFE, to make quantitative comparisons among these clones. Further, we have studied other malignant properties, such as s.c. tumour take, growth rate, host survival, and spontaneous metastatic ability, as well as biochemical determination of cellular DNA and protein content.

In the present communication these results are reported along with examination of the following: whether malignant properties vary simultaneously or separ-

Correspondence to: Norio Suzuki, Johns Hopkins Oncology Center, Radiobiology Laboratory, 600 N. Wolfe Street, Baltimore, Maryland 21205, U.S.A.

ately; whether variation of any malignant properties correlates with variation of DNA content and other cellular properties.

### MATERIALS AND METHODS

*Cells and culture.*—The fibrosarcoma clones were isolated from a methylcholanthrene-induced fibrosarcoma by repeated cloning in soft-agar medium for enhanced clonogenicity. From the initial cutlure (FSA1), 24 clones, designated FSA11 to FSA124, were isolated. FSA123, having the highest PE, was used for the second cloning, and 18 clones, FSA1231 to FSA12318, were established. The ranges of PE were $10^{-7}$–$10^{-6}$ at initial culture, $<0{\cdot}01$–$4{\cdot}8\%$ at the first cloning, and 1–35% at the second cloning. The PEs were heterogeneous among the clones. We have chosen clones of similar PE from the second cloning, but included clones of different DNA content for the analysis of the relationship between malignant characteristics and other cell properties. The parental fibrosarcoma cells, which had relative DNA content of 1·45 determined by flow microflurometry (FMF) is not included in these comparisons because the cells did not grow well *in vitro*. The cells have been stored in liquid $N_2$ to avoid unnecessary passages. For experiments the cells were cultured in a humidified $CO_2$ incubator with McCoy's 5A medium containing 20% foetal calf serum (Grand Island Biological Company, Grant Island, NY) (Suzuki & Withers, 1978). PE experiments were performed in medium containing 0·14% soft agar (Suzuki & Okada, 1976). Cells were inoculated in 4 or 8 tubes per clone, 100–500 cells per 5 ml medium.

*Cell counting and volume analysis.*—Cell count and volume distribution analyses were carried out with a model ZBI Coulter Counter and a Channelyzer II multichannel analyser and plotter (Coulter Electronics, Hialeah, FL). The counter was fitted with a 70$\mu$m diameter, 84$\mu$m long aperture. The system was calibrated with latex beads. The average cell volume in a given sample was calculated from the modal channel number of the volume distribution, in late-log-phase culture.

*Biochemical determination of cellular DNA and protein content.*—After 2 consecutive daily medium changes of the confluent culture, the cells were harvested by trypsinization and washed twice with Solution A and then once with saline by centrifugation. Although confluent cultures were used, the cells analysed biochemically were not a pure $G_1$ population, and hence these measurements were mean values for a mixture of $G_1$, S, and $G_2+M$ cells, while FMF determined the DNA content of $G_1$ cells. The DNA of $1$–$2\times10^6$ cells per ml was hydrolysed in 0·5N PCA by incubation at 70°C for 15 min, chilled in ice, and spun. The supernatants were used for DNA assay with diphenylamine (Burton *et al.*, 1956). The precipitates were solubilized in 1N NaOH at 70°C for 15 min and adjusted to $10^6$ cells per ml of 0·5N NaOH and served for protein measurements by Lowry's method.

*Lung colony formation, tumour take, and host survival.*—C3Hf/Kam male mice, 7–13 weeks old, were obtained from our specific-pathogen-free breeding colony and used in these experiments. For lung colony formation, single-cell suspensions of $10^5$ cells in 0·5 ml medium were injected into tail veins of unirradiated mice without addition of heavily irradiated cells or microspheres. Single-cell suspensions were obtained by trypsinization of late-log-phase cultures. These cultures were used as the standard condition because of the cycle dependence of lung colony formation (Suzuki *et al.*, 1977*a*). The cell suspensions were routinely examined by a phase-contrast microscope and a Coulter counter. There were no cell clumps in the suspensions used. This ease in making single-cell suspensions is one of the useful features of this FSA cell system (Suzuki & Withers, 1978). Mice were killed 19 days after injection. Lungs were placed in Bouin's fluid and lung colonies were scored macroscopically. Although the number of lung colonies of FSA1233 and FSA1231 reached their plateau level 10–12 days and 14 days after injection, respectively (Suzuki *et al.*, 1978) we chose a longer incubation time (19 days) because of possible differences of growth rate among clones.

For experiments involving tumour take or host survival, single-cell suspensions were made at $2\times10^5$/ml or $2\times10^6$/ml, unless otherwise specified. Mice were inoculated with 0·5 ml of suspension per site, 1 or 4 sites per mouse. Mice were observed daily.

*Spontaneous metastasis.*—In the experiment reported in Table III, $10^6$ FSA1231 or FSA1233 cells obtained by trypsinization of late-log-phase cultures were inoculated i.m. into the right thigh of each mouse. Mice were killed at 49, 56 and 63 days after irradiation.

FIG. 1.—Relationship between cellular DNA content determined by FMF and cell volume measured by a Coulter counter for various clones isolated from a single fibrosarcoma. Data for relative DNA content from Suzuki *et al.* (1977*b*).

FIG. 2.—Relationship between cell size and artificial lung-colony-forming efficiency. Mean ± s.e. among separate experiments. The numbers denote different clones of FSA123.

Tumour size was measured with calipers one day before killing, and is presented in Table III as the product of two diameters. In order to minimize loss of animals from local tumour growth, those killed for counting lung metastases at 49 and 56 days had the largest tumours at those times (Table III).

### RESULTS

PEs in percent (mean ± s.d. among 2–8 separate experiments) of the clones were: 19 ± 11 for FSA1231; 26 ± 18 for FSA1233; 13 ± 0·3 for FSA1235; 14 ± 5 for FSA1237; 33 ± 12 for FSA12311; 11 ± 6 for FSA-12316; and 6 ± 2 for FSA12318. Means of PEs of 4–8 tubes for each clone were calculated from each separate experiment and then pooled to obtain the mean ± s.d. among separate experiments for each clone.

Table I summarizes LCFE, cell volume, DNA content, and protein content of these clones. All parameters were heterogeneous

TABLE I.—*Lung-colony-forming efficiency, cell volume, DNA content, and protein content of each clone*

| FSA clone | Colonies per mouse* Mean ± s.e. | Cell volume† Mean ± s.e. | DNA content‡ (μg/10⁶ cells) Mean ± s.d. | Protein content§ (μg/10⁶ cells) Mean ± s.d. |
|---|---|---|---|---|
| 123·1 | 2·5 ± 1·6 (4)¶ | 1343 ± 39 (11)¶ | 14·4 ± 1·4 (3)¶ | 222 ± 13 (3)¶ |
| 123·3 | 30·4 ± 15·3 (6) | 2111 ± 49 (16) | 28·4 ± 2·0 (3) | 473 ± 32 (3) |
| 123·5 | 10·0 ± 1·9 (2) | 1445 ± 68 (3) | — | — |
| 123·7 | 1·2 ± 0·9 (7) | 1251 ± 33 (14) | 13·4 ± 1·3 (5) | 248 ± 46 (5) |
| 123·11 | 63·5 ± 11·3 (5) | 2449 ± 197 (2) | 27·2 ± 2·7 (3) | 442 ± 56 (3) |
| 123·16 | 0·15 ± 0·05 (2) | 1143 ± 27 (6) | 16·0 ± 0·8 (3) | 198 ± 18 (3) |
| 123·18 | 23·5 ± 9·5 (3) | 1925 ± 105 (2) | 19·3 ± 3·4 (4) | 385 ± 18 (4) |

* After i.v. injection of $10^5$ cells. into 10–20 mice per experiment.
† Determined by Coulter counter.
‡ Determined by diphenylamine method.
§ Determined by Lowry's method.
¶ Figures in parentheses show number of separate experiments.

TABLE II.—*Percentage s.c. tumour take**

| Cells/locus | Days after inoculation 15 days | 25 days | 50 days |
|---|---|---|---|
| FSA 1231—$10^6$ | 55 (22/40) | 82 (33/40) | 85 (34/40) |
| $10^6$ | 93 (37/40) | 93 (37/40) | 95 (38/40) |
| $10^5$ | 13 (2/15) | 20 (3/15) | 20 (3/15) |
| $10^5$ | 27 (4/15) | 47 (7/15) | 47 (7/15) |
| $10^5$ | 27 (4/15) | 33 (5/15) | 33 (5/15) |
| FSA 1233—$10^6$ | 98 (39/40) | 100 (40/40) | 100 (40/40) |
| $10^6$ | 100 (40/40) | 100 (40/40) | 100 (40/40) |
| $10^5$ | 70 (7/10)† | 60 (6/10) | 60 (6/10) |
| $10^5$ | 60 (6/10) | 80 (8/10) | 80 (8/10) |
| $10^5$ | 80 (8/10)† | 70 (7/10) | 70 (7/10) |

* In parentheses show no. of tumours/no. inoculation sites. Mice were inoculated at 4 loci per mouse at $10^6$ per locus or 1 locus per mouse at $10^5$ per locus. Summary of 10 experiments.

† In 2 mice, indefinite nodules were scored as positive at 15 days but were not palpable later.

FIG. 3.—Host-survival curves after s.c. inoculation of FSA1231 (○) or 1233 (●) cells, $10^6$/locus, 4 loci/mouse (data for 2 expts.).

TABLE III.—*Spontaneous lung metastases*

| Days* | Tumour Size† | No. mice‡ | Lung nodule bearer (%) | Nodules§ Mice with nodules | All mice |
|---|---|---|---|---|---|
| 1231 Cells | | | | | |
| 49 | 657 ± 11 | 16 | 11 (69) | 1·7 | 1·2 |
| 56 | 757 ± 17 | 16 | 9 (56) | 2·9 | 1·6 |
| 61 | 854 ± 22 | 20 | 15 (75) | 3·3 | 2·5 |
| 1233 Cells | | | | | |
| 49 | 954 ± 11 | 15 | 2 (13) | 1·5 | 0·2 |
| 56 | 1070 ± 27 | 15 | 3 (20) | 1·7 | 0·3 |
| 61 | — | 16 | 2 (13) | 1·0 | 0·1 |

* Mice were killed at these days after i.m. inoculation of $10^6$ cells.

† Products of 2 diam. of tumours 1 day before killing. Mean ± s.e. ($mm^2$).

‡ Mice killed were those bearing the largest tumours at the time.

§ Mean nodule number per mouse.

among these clones from the same original tumour.

The relationship between cell volume and DNA content, determined by biophysical methods, is shown in Fig. 1. The results of Fig. 1 and Table I show that the 3 parameters are correlated.

Fig. 2 shows that LCFE is correlated with cell volume.

Tumour transplantability and host survival time after s.c. injection of FSA1231 or FSA1233 are summarized in Table II and Fig. 3. The times to 50% survival after tumour-cell implantation, in two separate experiments, were 33 and 39 days for FSA1233, and 58 and 61 days for FSA1231 (Fig. 3). These data show that FSA1233, the clone of larger cells with more DNA, was more malignant in terms of the three tests than was FSA1231, the clone of smaller cells with less DNA.

Table III presents spontaneous metastasis frequency to the lung from i.m. inoculated FSA1231 or FSA1233 cells. FSA1231 developed spontaneous metastasis in 56–75% of mice, thereas FSA1233 did so in only 13–20% of mice, indicating that the smaller-cell tumour, FSA1231, is more efficient in spontaneous lung metastasis from leg tumours, even though it was less efficient than the large-cell, FSA1233, in producing lung nodules after i.v. injection, and slower in its local growth in the thigh.

### DISCUSSION

Most tumour cells are aneuploid or heteroploid, and also correspondingly higher and heterogeneous in DNA content (Atkin, 1966; Stich, 1960). Karyotype analyses have also provided supportive evidence for the concept that tumour cells vary and evolve (Foulds, 1969; Hauschka, 1961; Hsu, 1961; Makino, 1957; Medina, 1975; Nowell, 1976). One of the purposes of the present study was to inquire why the DNA content of most tumour cells is increased, and to examine whether there is any positive role of increase in DNA content or alteration of DNA content in the development of malignant properties.

The clones were isolated after repeated clonings in soft agar medium, and have been kept in liquid $N_2$ except for experimental use. Since we have assumed that malignancy involves multiple factors, minimizing the passage of daughter clones should have reduced the variations introduced experimentally. Cloning in soft agar was used to eliminate or lessen "noise" in the intercomparisons of tumour-cell properties that would result from contamination by nonclonogenic cells, nonmalignant variants or normal cells.

The present study showed that DNA content was correlated with cellular protein content and cell size, and these correlated with LCFE, s.c. tumour take rate, and subsequent growth rate and host survival. In local growth ability and LCFE after i.v. injection of tumour cells, a large-cell clone was more efficient than a small-cell clone. However, with respect to "spontaneous" development of (lung) metastases, from a tumour implanted and growing in the thigh, FSA1231, a small-cell clone, was more efficient.

The DNA content varied concomitantly with cell volume, protein content, and the malignant characteristics of the clones in terms of local growth ability. Therefore, the increase of DNA content appears to be significantly involved in the process that leads to variation in malignant characteristics, although the close correlation of the phenomena is not enough to establish a causal relationship. In addition, the close relationship between DNA content and protein content is not unique only to this sytem, and the phenomenon has been documented, for example, in mouse mammary carcinoma, Ehrlich ascites tumour, and lymphoma cells of different ploidy (Bassleer & De Paermentier, 1977; Kit, 1960).

The reasons for the increased growth ability of the cells with increased DNA content or cell volume remain to be clarified. It has been described that near-tetraploid tumour cells were more efficient than near-diploid tumour cells in acquiring strain independent transplantability, *i.e.* against immunogenic host reaction (Hauschka & Levan, 1953). In cultured mammalian cells, increased resistance to drugs or hypertonicity paralleled gene amplification (Alt *et al.*, 1978) or polyploidization (Li *et al.*, 1978). All the clones of the present system so far examined had similar or higher levels of DNA content relative to the parental fibrosarcoma (Suzuki *et al.*, 1977*b*). This indicates that increasing DNA content was closely related to the process of increasing clonogenicity in adverse medium from an order of $10^{-7}$ to $10^{-6}$ to a level of 1–35%. During developmental and evolutionary processes in normal cells, the DNA content and cell volume maintain a constant ratio (Biodsky & Uryvaena, 1977; Szarski, 1976) as was the case in this fibrosarcoma system. Polyploidization or increasing DNA content may enhance the survivability and growth ability of neoplastic cells under an adverse environment in a similar sense to that postulated for the increased efficiency of normal cells of higher ploidy in developmental and evolutionary processes. This preferential growth ability of part of a tumour-cell population in a certain environment may be an important factor in a selective process during neoplastic development.

Lung-colony-forming efficiency is also higher for S or $G_2+M$ cells than for $G_1$ cells of the FSA1233 cell (Suzuki *et al.*, 1977*a*). One explanation for the increased LCFE of large cells is that they are trapped more efficiently in lung capillaries; however, such an explanation could not be applied to the increased efficiency of s.c. growth of cells from the larger-cell clone reported here.

Fidler (1973) selected cell lines with enhanced LCFE from B16 melanoma by repeating lung-colony formation many times. Organ selectivity in the metastatic process, and heterogeneity of metastatic potential among tumour cells have been advanced as essential factors in metastasis (Briles & Kornfeld, 1978; Brunson *et al.*, 1978; Fidler & Kripke, 1977; Nicolson & Winkelhake, 1975; Suzuki *et al.*, 1978*a*; Susuki & Withers, 1978, 1979; Tao & Burger, 1977; Tao *et al.*, 1979; Tarin & Price, 1979). The organ selectivity and increased metastatic potential has been ascribed to selective adhesion of the tumour cells to a certain tissue or organ (Nicolson & Winkelhake, 1975; Brunson *et al.*, 1978).

While our experiments support heterogeneity of tumour cells as an essential part of metastatic growth, selective adhesion of the tumour cells to a certain tissue or organ cannot explain the differences seen between FSA1231 and FSA1233 observed in LCFE and spontaneous metastasis frequency. If the affinity of tumour cells for the lung is the prime determinant of these differences, the clone FSA1233, which had a higher LCFE than FSA1231, would have a higher frequency of spontaneous metastasis to the lung. The important factor(s) in this system remain to be clarified, although the metastatic process requires other factors than those involved in local growth (Suzuki & Withers, 1979; present results).

In conclusion, cellular DNA content, volume, protein content, and distant metastasis, as well as LCFE, s.c. tumour transplantability, and growth rate, were heterogeneous among clones derived from a single murine fibrosarcoma. Malignancy can vary and was not "all or none" in its nature. Increasing DNA content was an important factor in the development and maintenance of malignancy in terms of local growth ability, but the development of spontaneous distant metastasis seemed to involve different factor(s).

This investigation was supported in part by Grants Number CA-11138 and CA-06294, awarded by the National Cancer Institute, DHEW.

Animals used in this study were maintained in facilities approved by the American Association for Accreditation of Laboratory Animal Care, and in accordance with current regulations and standards of the United States Department of Agriculture and Department of Health, Education, and Welfare, National Institutes of Health.

We would like to thank Mrs R. Goddard and Ms A. McCarver for typing the manuscript.

### REFERENCES

ALT, F. W., KELLEMS, R. E., BERTINO, J. R. & SCHIMKE, R. T. (1978) Selective multiplication of dihydrofolate reductase genes in methotrexate resistant variants of cultured murine cells. *J. Biol. Chem.*, **253**, 1357.

ATKIN, N. B., MATTINSON, G. & BAKER, M. C. (1966) A comparison of the DNA content and chromosome number of fifty human tumours. *Br. J. Cancer*, **20**, 87.

BASSLEER, R. & DE PAERMENTIER, F. (1977) Cytotological and cytochemical analysis of two mouse cancer cell lines. Caryotype, number of nucleoli, DNA, RNA and protein contents. *Eur. J. Cancer*, **13**, 589.

BIODSKY, W. Y. & URYVAENA, I. V. (1977) Cell polyploidy: Its relation to tissue growth and function. *Int. Rev. Cytol.*, **50**, 275.

BRILES, E. B. & KORNFELD, S. (1978) Isolation and metastatic properties of detachment variants of B16 melanoma cells. *J. Natl Cancer Inst.*, **60**, 1217.

BRUNSON, K. W., BEATTIE, G. & NICOLSON, G. L. (1978) Selection and altered properties of brain-colonizing metastatic melanoma. *Nature*, **272**, 543.

BURTON, K. (1956) A study of the condition and mechanism of the diphenylamine reaction for the colorimetric estimation of deoxyribonucleic acid. *Biochem. J.*, **62**, 315.

FIDLER, I. J. (1973) Selection of successive tumor lines for metastasis. *Nature* (*New Biol.*), **242**, 148.

FIDLER, I. J. & KRIPKE, M. L. (1977) Metastasis results from pre-existing variant cells within a malignant tumor. *Science*, **197**, 893.

FOULDS, L. (1969) *Neoplastic Development*, Vol. 1. London: Academic Press. p. 41.

HAUSCHKA, T. S. (1961) The chromosomes in ontogeny and oncogeny. *Cancer Res.*, **21**, 957.

HAUSCHKA, T. S. & LEVAN, A. (1953) Inverse relationship between chromosome ploidy and host-specificity of sixteen transplantable tumors *Exp. Cell. Res.*, **4**, 457.

HSU, T. C. (1961) Chromosomal evolution in cell populations, *Int. Rev. Cytol.*, **12**, 69.

KIT, S. (1960) Nucleic acid synthesis in the neoplastic cell and impact of nuclear changes on the biochemistry of tumor tissue: A review. *Cancer Res.*, **20**, 1121.

Li, C. C., Karnovsky, M. J., Lin, P. S. & Lin, E. C. C. (1978) The selection of a stable rat hepatoma variant with concomitant increase in ploidy and permeability to glycerol. *J. Cell. Physiol.*, **94**, 197.

Makino, S. (1957) The chromosome cytology of the ascites tumors of rats with special reference to the concept of the stem cell. *Int. Rev. Cytol.*, **6**, 26.

Medina, D. (1975) Tumor progression. In *Cancer*, Vol. 3 (Ed. Becker). New York: Plenum. p. 99.

Nicholson, G. L. & Winkelhake, J. L. (1975) Organ Specificity of blood-borne tumor metastasis determined by cell adhesion? *Nature*, **255**, 230.

Nowell, P. C. (1976) The clonal evolution of tumor cell populations. *Science*, **194**, 23.

Stich, H. F. (1960) The DNA content of tumor cells, II. Alterations during the formation of hepatomas in rats. *J. Natl Cancer Inst.*, **24**, 1283.

Suzuki, N. & Okada, S. (1976) Isolation of nutrient deficient mutants and quantitative mutation assay by reversion of alanine-requiring L5178Y cells. *Mutat. Res.*, **34**, 489.

Suzuki, N., Frapart, M., Grdina, D. J., Meistrich M. L. & Withers, H. R. (1977*a*) Cell cycle dependency of metastatic lung colony formation. *Cancer Res.*, **37**, 3690.

Suzuki, N., Withers, H. R. & Lee, L. Y. (1977*b*) Variability of DNA content of murine fibrosarcoma cells. *Nature*, **269**, 531.

Suzuki, N. & Withers, H. R. (1978) Isolation from a murine fibrosarcoma of cell lines with enhanced plating efficiency *in vitro*. *J. Natl Cancer Inst.*, **60**, 179.

Suzuki, N., Withers, H. R. & Williams, M. (1978) Heterogeneity and variability of artificial lung colony forming ability among clones from mouse fibrosarcoma. *Cancer Res.*, **38**, 3349.

Suzuki, N. & Withers, H. R. (1979) Lung colony formation: A selective cloning process for lung-colony-forming ability. *Br. J. Cancer*, **39**, 196.

Szarski, J. (1976) Cell size and nuclear DNA content in vertebrates. *Int. Rev. Cytol.*, **44**, 93.

Tao, T. W. & Burger, M. M. (1977) Non-metastasizing variants selected from metastasizing melanoma cells. *Nature*, **270**, 437.

Tao, T. W., Matler, A., Vogel, K. & Burger, M. M. (1979) Liver-colonizing melanoma cells selected from B-16 melanoma. *Int. J. Cancer*, **23**, 854.

Tarin, D. & Price, J. E. (1979) Metastatic colonization potential of primary tumour cells in mice. *Br. J. Cancer*, **39**, 740.